# Fecal PCR survey and genome analysis of *Lawsonia intracellularis* in China

**DOI:** 10.3389/fvets.2024.1324768

**Published:** 2024-02-07

**Authors:** Lei Wang, Wenqing Wu, Lifeng Zhao, Zhanwei Zhu, Xinzhi Yao, Jie Fan, Hongjian Chen, Wenbo Song, Xi Huang, Lin Hua, Ping Qian, Huanchun Chen, Zhong Peng, Bin Wu

**Affiliations:** ^1^State Key Laboratory of Agricultural Microbiology, College of Veterinary Medicine, Huazhong Agricultural University, Wuhan, China; ^2^The Cooperative Innovation Center for Sustainable Pig Production, Huazhong Agricultural University, Wuhan, China; ^3^Guangxi YangXiang Co., Ltd., Guigang, China; ^4^College of Informatics, Hubei Key Laboratory of Agricultural Bioinformatics, Huazhong Agricultural University, Wuhan, China; ^5^Hubei Hongshan Laboratory, Wuhan, China

**Keywords:** *Lawsonia intracellularis*, TaqMan qPCR, screening, pig farms, complete genome sequence

## Abstract

Proliferative enteropathy caused by *Lawsonia intracellularis* is an important economic associated disease to pig industry, but the knowledge about the prevalence of *L. intracellularis* in pig farms in China is limited. In addition, there is no complete genome sequence available for *L. intracellularis* isolates from China. In this study, we developed a TaqMan qPCR for the screening of *L. intracellularis* by targeting the bacterial 16S rDNA gene. Laboratory evaluations revealed a good sensitivity and specificity on detecting *L. intracellularis* nucleic acid. Using this method, we investigated 891 fecal samples from apparently healthy pigs in 47 farms. The results demonstrated a screening positive rate of 37.3% (95% CI, 34.1–40.5%) for the samples, and a farm screening positive rate of 93.6% (95% CI, 65.3–94.4%). The screening positive rate at herd level ranged from 6.67% (95% CI, 0.2–31.9%) to 40% (95% CI, 38–79.6%), while at animal level, the highest screening positive rate was found in 12-week-old pigs [85.7% (95% CI, 67.3–96.0%)]. Investigation of 705 diarrheal or bloody feces from symptomatic pigs revealed that the highest positive rate was found in replacement gilts which was 37.18% (95% CI, 45.1–89.5%). Secondly, we conducted the complete genome sequence of a *L. intracellularis* PPE-GX01-2022 from China through PacBio sequencing. The genome of PPE-GX01-2022 consisted of a chromosome of 1,439,110 bp in length and three plasmids of 193,063, 39,799, and 27,067 bp, respectively. This genome encoded 1,428 predicted proteins, 44 tRNAs, and 6 rRNAs. Sequence comparisons demonstrated that the genome sequence of PPE-GX01-2022 was highly homologous to those of two isolates from US, and these three isolates shared 1,378 core genes. The screening results suggest a high prevalence rate of *L. intracellularis* in Chinese pig farms. In addition, the genome sequence of the Chinese isolate was highly homologous to those of the field isolates from the US.

## Background

The gram-negative microaerophilic obligate intracellular bacterium *Lawsonia intracellularis* can cause proliferative enteropathy (PE) in weaned and growing pigs <4 months, which is associated with decreased weight gain and low mortality; it can also lead to hemorrhagic enteritis (HE) in mature pigs older than 4 months, which is characterized by intestinal hemorrhages and sudden death (1). *L. intracellularis* is prevalent worldwide and has caused big economic losses. For example, it estimates that PE leads to an increase cost of 2–20 dollars of feed consumption in every diseased-pig and causes annual economic losses of $20 million to the pig industry of the United States as well as £2–£4 million to the pig industry of the United Kingdom (2). However, many knowledge gaps on the biology, epidemiology, and pathogenesis of the bacterium remain to be bridged. For example, there are only eight whole genome sequences of *L. intracellularis* publicly available on NCBI GenBank database, and only two of them are complete genome sequences (3). Seven of these isolates are from pigs (three from Japan, one from South Korea, one from United Kingdom, two from United States) and the remaining one is recovered from a horse in United States. The genome sizes of the two complete sequences (N343, GenBank accession. CP004029; PHE/MN1-00, GenBank accession. AM180252) are 33.09 Mb, while those of the remaining six are 32.90 Mb, respectively. A lack of more genome sequences of *L. intracellularis* from different parts of the world limits the further understanding of the genetic evolutionary characteristics of the bacterium.

Serological methods particular detection of antibodies against *L. intracellularis* through enzyme linked immunosorbent assay (ELISA) are commonly applied for monitoring the prevalence of *L. intracellularis* infection in clinical investigations (1, 4). For example, a study investigated the seroprevalence of *L. intracellularis* in pigs at different ages in 134 farm sites in 1998 and 43 farm sites in 2008 in Great Britain and the Republic of Ireland (5). Both two surveys in this study revealed higher than 90% seropositive pigs at 20–23 weeks of age on British farms (97.8% in 1998; 93.1% in 2008) and Irish farms (97% in 1998; 92.9% in 2008) (5). In another serological investigation conducted on farms in France and Spain, postweaning pigs on 29 French farms (33 total) and 20 Spanish farms (29 total) had a pattern of infection characterized by seroconversion in the grower period, representing a respective farm-seropositivity of 87.88 and 68.97% (6). In addition to serological methods, several molecular methods have been also developed for detecting *L. intracellularis* DNA in tissue specimens or fecal samples, and real-time quantitative PCR (qPCR) is the mostly-notable one (1, 4). A couple of previous studies reported the development of qPCR to detect *L. intracellularis* DNA based on different target genes, including *ubiE* (7), the 16S ribosomal RNA (rRNA) gene (8, 9), *aspA* (10, 11), and the 16S ribosomal DNA (rDNA) gene (12, 13). While these methods demonstrate good diagnostic specificity and sensitivity under laboratory validation, there are not used in clinical investigations as field detection is beyond these studies. However, there are still studies involve in the application of qPCR for clinical investigation. For example, a study investigated 144 herds in five European countries (Germany, Denmark, Spain, the Netherlands and the United Kingdom) by examination of 6,450 fecal samples using commercial qPCR kits (“Kylt^®^ PIA [*Lawsonia intracellularis*]” and “Kylt^®^ Quantitative standard for *Lawsonia intracellularis*”, AniCon Labor GmbH, Hoeltinghausen, Germany) and demonstrated a herd prevalence of 90.3% (79.2–100.0%) among the tested herds (14). All the above findings suggest a high prevalence of *L. intracellularis* on pig farms around the world.

China is the largest pig rearing and pork producing country in the world. However, current data on the prevalence of *L. intracellularis* on Chinese pig farms is still limited. A recent study investigated 3,586 serum samples in eight major pig-producing provinces during 2019–2020 and revealed that 2,837 (79.1%, 95% CI: 77.7–80.4%) were seropositive for *L. intracellularis* (15). In an earlier study, examination of 1,060 serum samples from 14 commercial farms through China revealed a 57% (95% CI: 50–64%) seroprevalence of *L. intracellularis* (16). These sero-epidemiological studies suggest a high prevalence of *L. intracellularis* in Chinese pig farms. However, it is still lack of much data on the prevalence of *L. intracellularis* from the perspective of etiology. In this study, we developed a TaqMan qPCR to detect *L. intracellularis* DNA from porcine fecal samples. The objective is to investigate the prevalence of *L. intracellularis* in several Chinese pig farms by detecting bacterial DNA using qPCR. In addition, we also aim to generate a complete genome sequence of a Chinese *L. intracellularis* isolate from clinical samples to help understand the genomic characteristics of *L. intracellularis* prevalent in China.

## Results

### Development of a TaqMan qPCR for the detection of *L. intracellularis*

A TaqMan qPCR for detecting *L. intracellularis* was developed based on the 16S rRNA gene (GenBank accession no. U30147), following the protocol described previously (13). Optimization of the amplification conditions revealed that an optimal reaction occurred when the concentrations of the forward and reverse primers set as 0.5 μM while the concentration of the probe set as 0.25 μM. The limit of the qPCR method detecting the standard plasmid was 36 copies/μl (Table 1). Construction of the standard curves demonstrated that there was a strong linear correlation (*R*^2^ = 0.9999; y = −3.547lgx+40.520) between the Ct values and the corresponding copy numbers of the 16S rDNA gene of *L. intracellularis*. Evaluation of specificity showed that the qPCR method only gave amplification curve to *L. intracellularis* (Supplementary Figure 1). The coefficient of variation (CV) values of the method detecting different concentrations of the standard plasmids were lower than 1.5 (Table 2). There was no difference (χ^2^ = 1, *p* > 0.05) on the true positive rate of clinical samples detected by the qPCR method developed in this study and the qPCR method reported previously (17) (Table 3). The detection limit of the qPCR method was also similar to that of the previously reported method (36 vs. 30 copies/μl).

**Table 1:** Limits of the TaqMan qPCR method detecting the standard plasmid.

**Dilution folds**	**Plasmid concentrations (ng/μl)**	**DNA copies (copies/μl)**	**CT values**
10^−2^	412.2	6.73 × 10^9^	14.103
10^−3^	412.2 × 10^−2^	6.73 × 10^8^	17.343
10^−4^	412.2 × 10^−3^	6.73 × 10^7^	20.943
10^−5^	412.2 × 10^−4^	6.73 × 10^6^	24.517
10^−6^	412.2 × 10^−5^	6.73 × 10^5^	28.243
10^−7^	412.2 × 10^−6^	6.73 × 10^4^	31.977
10^−8^	412.2 × 10^−7^	6.73 × 10^3^	34.962
10^−9^	412.2 × 10^−8^	6.73 × 10^2^	No detection result
10^−10^	412.2 × 10^−9^	6.73 × 10	No detection result
Nucleotide-free water	0	0	No detection result

**Table 2:** Repeatability of the TaqMan qPCR method detecting the standard plasmid.

**Dilutes**	**Repeats**			**Mean ±standard deviation**	**Coefficient of variation (CV) values**
	**1**	**2**	**3**		
6.73 × 10^8^	18.63	19.02	18.51	18.72 ± 0.267	1.42
6.73 × 10^7^	21.85	21.85	21.85	21.89 ± 0.075	0.34
6.73 × 10^6^	24.75	24.75	24.66	24.69 ± 0.049	0.19

**Table 3:** Comparison of the TaqMan qPCR method and a previously reported qPCR (China AQSIQ) on detecting clinical samples.

**China AQSIQ**	**TaqMan qPCR**		**Total**	**Statistical analysis**
	**Numbers of positive samples**	**Numbers of negative samples**		
Numbers of positive samples	18	0	18	
Numbers of negative samples	0	50	50	χ^2^= 1, *p* > 0.05
Total	18	50	68	

### Fecal PCR investigation of *L. intracellularis* in pig farms in China

**Figure 1:**
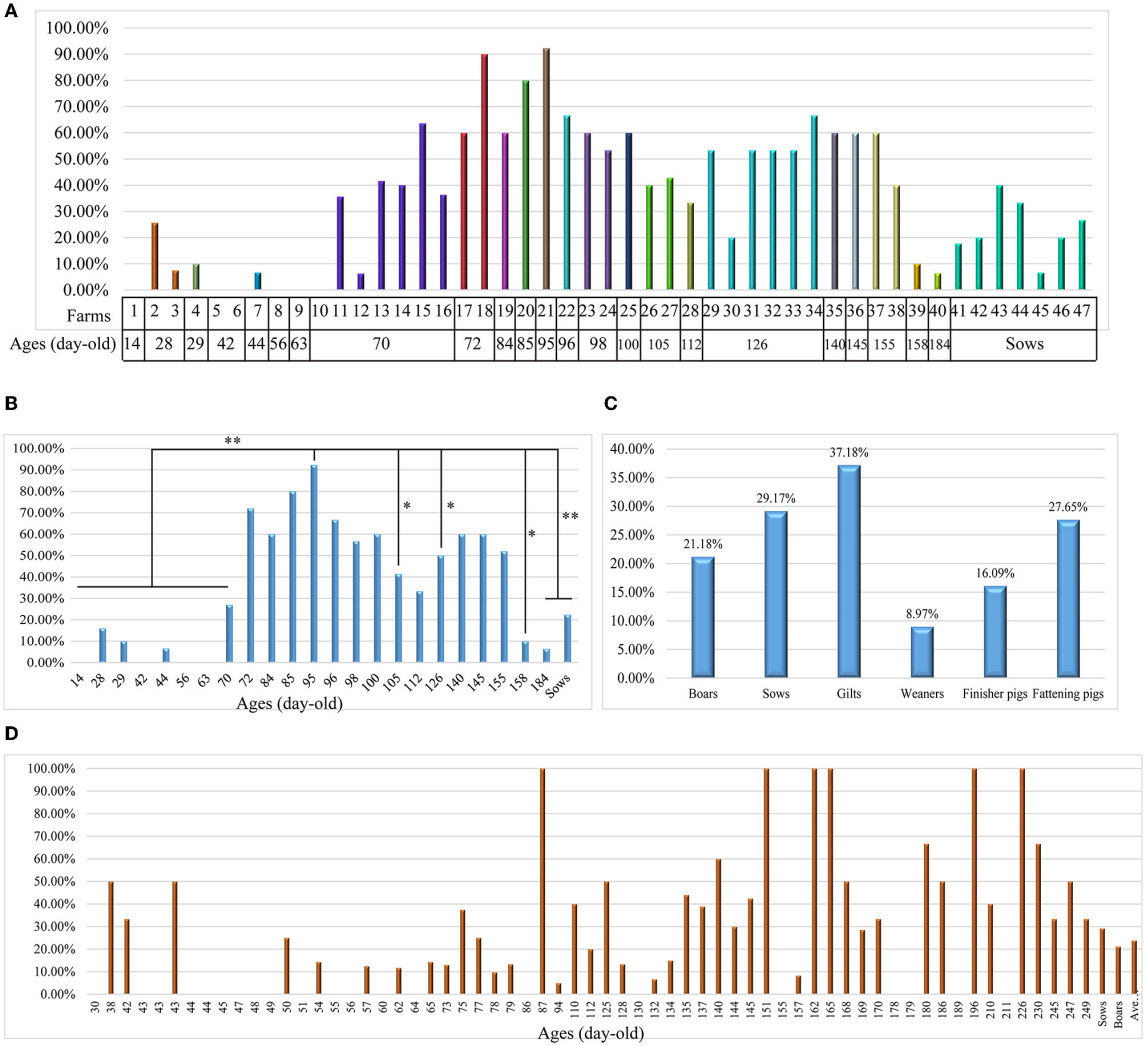
Investigation of *Lawsonia intracellularis* from clinical samples using the TaqMan qPCR developed in this study. **(A)** A column chart showing the screening rates of *L. intracellularis* from 891 fecal samples from healthy pigs at different ages in 47 farms in China; **(B)** A column chart showing the screening rates of *L. intracellularis* from feces of healthy pigs at different ages; statistical significance (Chi-square test; *P* < 0.05) was analyzed based on the comparisons of positive rates investigated from fecal samples collected from pigs aged at different days; **(C)** A column chart showing the screening rates of *L. intracellularis* from 705 diarrheal or bloody feces from symptomatic pigs belonging to different groups; **(D)** A column chart showing the screening rates of *L. intracellularis* from feces of symptomatic pigs at different ages. ^*^p < 0.5; ^**^p < 0.01.

To understand the prevalence of *L. intracellularis* on Chinese pig farms, we collected 891 fecal samples from apparently healthy pigs in 47 farms for bacterial DNA screening. The results revealed that 37.3% (95% CI: 34.1–40.5%) of the samples were positive for *L. intracellularis* DNA, with a farm positive rate of 93.6% (95% CI: 65.3–94.4%). High positive screening rates were observed in pigs aged between 72- and 155-day-old (Figures 1A, B). In particular, the highest positive investigation rate (92.31%) was found in pigs at 95 days of age (Figures 1A, B). We next investigated 705 diarrheal or bloody feces from different pig herds (boars, sows, gilts, weaners, finisher pigs and fattening pigs) delivered from 125 pig farms (Supplementary Table 2). The results demonstrated the positive screening rates among different herds ranging from 8.97 to 37.18% (Figure 1C). Regarding diarrheal or bloody samples from pigs at different ages, highest positive screening rates were found in pigs at 87-, 151-, 162-, 165-, 196-, and 226 days of age (Figure 1D).

### Generation of the first complete genome sequence of *L. intracellularis* from China

**Figure 2:**
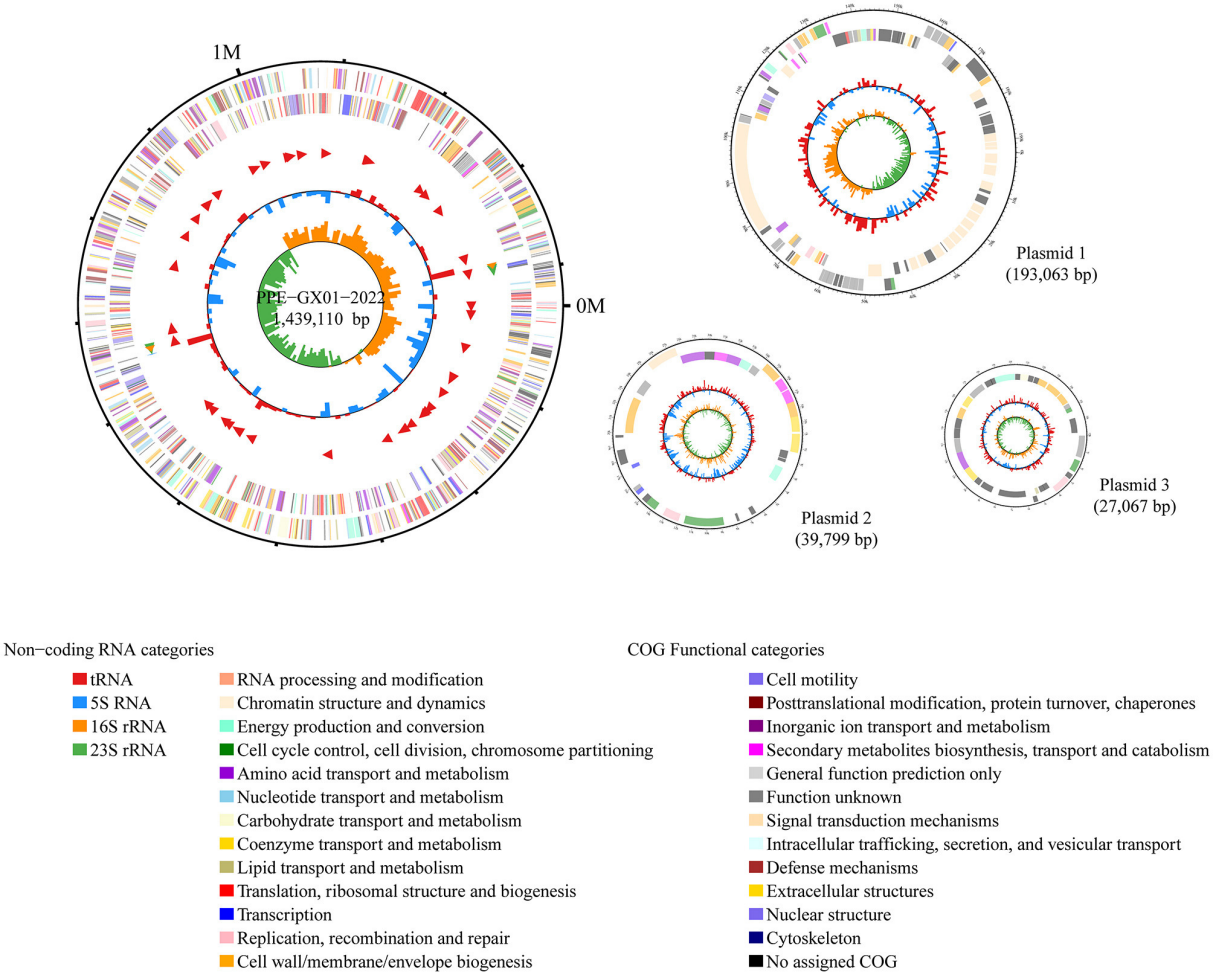
Circle maps of the complete genome sequence of *L. intracellularis* PPE-GX01-2022. The figure shows the chromosomal genome as well as the three plasmids harbored the PPE-GX01-2022. Distribution of RNAs and genes as well as the COG functions of these genes are given at the bottom of the figure.

The complete genome sequence of a *L. intracellularis* isolate, designated PPE-GX01-2022, was generated using PacBio sequencing. The genome of this isolate consisted of a chromosome of 1,439,110 bp in length and three plasmids of 193,063 bp (GX01-2022-P1), 39,799 bp (GX01-2022-P2), and 27,067 bp (GX01-2022-P3), respectively (Figure 2). Gene annotation revealed that the complete genome sequence of PPE-GX01-2022 encoded 1,428 predicted proteins, as well as 44 tRNAs, 6 rRNAs (5S rRNA, 2; 16S rRNA, 2; 23S rRNA, 2), and 5 other non-coding RNAs (ncRNAs; Table 4). No CRISPR elements or prophage regions were identified in the bacterial genome. Prediction of antimicrobial resistance genes (ARGs) demonstrated that PPE-GX01-2022 contained 27 genes conferring resistance to macrolides (seven genes), peptide antibiotics (five genes), fluoroquinolones (three genes), glycopeptide antibiotics (two genes), isoniazid (two genes), aminoglycosides (one gene), elfamycins (one gene), fosfomycin (one gene), fusidic acids (one gene), monobactams (one gene), mupirocin (one gene), pyrazinamide (one gene), and sulfonamides (one gene; Supplementary Table 3). However, prediction of virulence factor genes (VFGs) only identified two VFGs in the genome of PPE-GX01-2022 (VFG046458 associated with bacterial adherence and invasion; VFG002891 similar to bacterial O-antigen; Supplementary Table 4). Notably, none of these ARGs or VFGs were situated on the plasmids.

**Table 4:** General features of the complete genome sequences of *L. intracellularis* isolates from swine.

**Strains**	**PPE-GX01-2022**	**N343**	**PHE/MN1-00**
GenBank accession numbers		NC_020127	NC_008011
Total genome sizes (bp)	1,699,039	1,719,192	1,719,014
GC Content (%)	32.93	33.09	33.09
Number of plasmids	3	3	3
Size of chromosome (bp)	1,439,110	1,457,568	1,457,619
Size of plasmid 1 (bp)	193,063	194,613	194,553
Size of plasmid 2 (bp)	39,799	39,878	39,794
Size of plasmid 3 (bp)	27,067	27,133	27,048
CDSs	1,428	1,432	1,432
tRNAs	44	44	45
rRNAs	6	6	6
Other non-coding RNAs	5	4	4
CRISPR elements	0	0	0
Prophages	0	0	0
**Average nucleotide identity**			
PPE-GX01-2022	100%	99.93%	99.93%
N343	99.93%	100%	99.99%
PHE/MN1-00	99.93%	99.99%	100%

**Figure 3:**
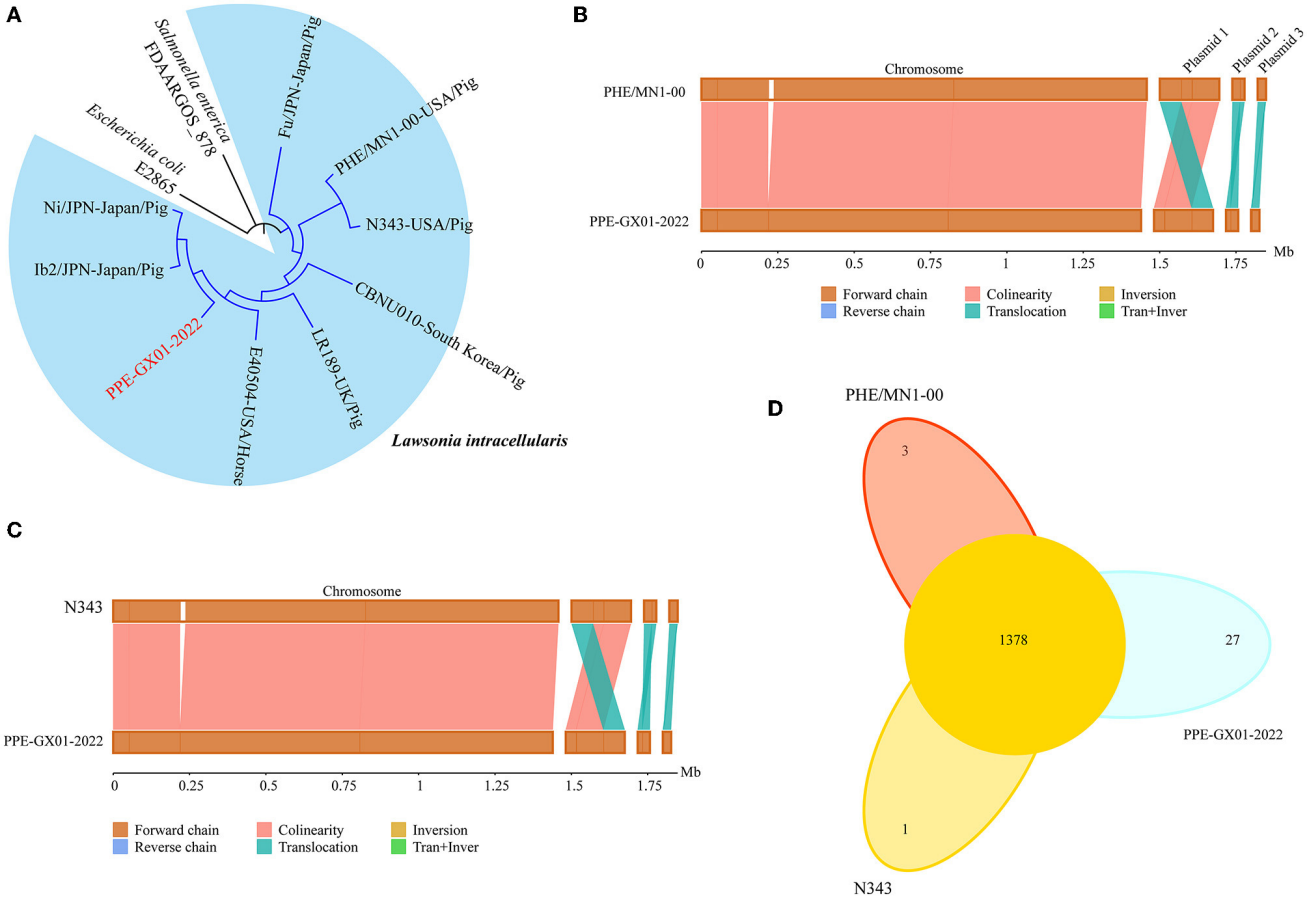
Comparative genomics of *L. intracellularis* isolates PPE-GX01-2022, PHE/MN1-00, and N343. **(A)** A maximum likelihood tree of PPE-GX01-2022 and the other *L. intracellularis* isolates, as well as a *E. coli* strain and *Salmonella* strain constructed using RAxML; **(B)** Sequence comparisons between the chromosomal genomes and the plasmids of PPE-GX01-2022 to that of PHE/MN1-00; **(C)** Sequence comparisons between the chromosomal genomes and the plasmids of PPE-GX01-2022 to that of N343; **(D)** A Venn diagram demonstrates the numbers of shared genes and isolate-specific genes among PPE-GX01-2022, PHE/MN1-00, and N343.

Phylogenetic analysis using the whole genome sequence data revealed that PPE-GX01-2022 was closely related to *L. intracellularis* isolates from the other regions in the world (Figure 3A). Since the currently available commercial PPE attenuated vaccine (Enterisol^®^Ileitis; Boehringer Ingelheim) was developed based on a US clinical isolate, we compared the complete genome sequence of PPE-GX01-2022 against those of the two US clinical isolates N343 (GenBank accession no. NC_020127) and PHE/MN1-00 (GenBank accession no. NC_008011), respectively (Figures 3B, C). The results demonstrated that the genome sequence of PPE-GX01-2022 shared a 99.93% average nucleotide identity to those of N343 and PHE/MN1-00 (Table 4). Interestingly, the genome sequences of the three plasmids carried by PPE-GX01-2022 were also highly homologous (average nucleotide identity > 99.9%) to those of the plasmids carried by N343 and PHE/MN1-00, respectively. Pan-genome analysis revealed that *L. intracellularis* isolates PPE-GX01-2022, N343, and PHE/MN1-00 shared a total of 1,378 core genes as well as 27, 1, and 3 isolate-specific genes, respectively (Figure 3D; Supplementary Table 5). Most of the specific genes carried by PPE-GX01-2022 encoded hypothetic proteins (16 genes), and the remaining 11 genes encoded proteins participating in translation, ribosomal structure and biogenesis (three genes), carbohydrate transport and metabolism (two genes), inorganic ion transport and metabolism (two genes), energy production and conversion (one gene), replication, recombination and repair (one gene), cell wall/membrane/envelope biogenesis (one gene), and function unknown (one gene), according to the clusters of orthologous groups of proteins (COG) functional analysis (Supplementary Table 5).

## Discussion

*L. intracellularis* is an obligately intracellular bacterium and is very difficult to be routinely cultured *in vitro*; therefore, the confirmation of *L. intracellularis* infection mainly relied on several alternative methods instead of bacterial culture (1). Amongst using laboratory-based PCR assays confirming *L. intracellularis* DNA in fecal or intestinal mucosal samples is one of the widely used method (4). In this study, we developed a TaqMan qPCR for detecting *L. intracellularis* by targeting the bacterial 16S rDNA gene. It is worthy of note that the 16S rDNA gene is a target commonly used for the development of DNA-based diagnostic methods of *L. intracellularis* infection in many studies (13, 18–20). A laboratory evaluation revealed that the method developed in this study displayed good specificity on detecting *L. intracellularis*, and in particular, the qPCR results for detecting clinical samples were identical with those detected by a previously published qPCR method (17). These findings suggest that the qPCR method we developed in this study could be applied as an effective alternative method for *L. intracellularis* detection in clinical investigations.

In this study, we investigated two groups of samples. The first group included fecal samples randomly collected from healthy pigs at different ages in 47 Chinese pig farms, and the investigation revealed a high farm positive rate of *L. intracellularis* [93.6% (95% CI: 65.3–94.4%)]. While few studies have reported the prevalence of *L. intracellularis* based on the detection of bacterial DNA on pig farms in China, two seroprevalence studies using ELISA assays detecting specific antibodies against *L. intracellularis* have demonstrated a high bacterial seropositivity (over 70%) (15, 16). From another article published in Chinese, the farm positive rate of *L. intracellularis* on 21 pig farms in China was as high as 100% (21). The above findings suggest that the prevalence of *L. intracellularis* infections might be common on Chinese pig farms, and more attentions should be paid on the prevention and control of the disease. This might be very important as the Chinese government issued a policy (Ministry of Agriculture and Rural Affairs Announcement No. 194, 07-10-2019) banning the addition of antibiotics to feed to promote animal growth on July 1st, 2020, for the purpose of combating the global rapid increasing of antimicrobial resistance. It is noteworthy that the infection rate of *L. intracellularis* at farm level in several other regions in the word is also high. For example, a previous study investigated the prevalence of *L. intracellularis* infection in 65 Korean herds through indirect immunofluorescence antibody technique and found an infection rate of 100% on pig farms (22). A more recent epidemiological study revealed a positive rate of 90.3% (79.2–100.0%) at farm level in six European countries (Germany, Denmark, Spain, the Netherlands and the United Kingdom) by detecting *L. intracellularis* DNA from fecal samples using qPCR (14).

We also investigated another group of samples in this study. This group of samples included diarrheal and bloody feces from different pig herds (boars, sows, gilts, weaners, finisher pigs and fattening pigs), and these samples were delivered by 125 farms for *L. intracellularis* diagnosis. Investigation on this group of samples demonstrated an average positive rate of 23.83%. This value is also similar to positive rate of a recent epidemiological study performed in six European countries by detecting *L. intracellularis* DNA from fecal samples from diarrheal pigs using qPCR [26.2% (15.9–41.5%)] (14). The above findings indicate that diarrhea caused by *L. intracellularis* should not be ignored. In addition, our screening of fecal samples from both healthy pigs and/or diarrheal pigs demonstrated a higher positive rate in old pigs than in young pigs. This result agrees well with the report that *L. intracellularis* affects more frequently post-weaned pigs between 6 and 20 weeks of age (1, 23). It is noteworthy that several published studies investigating the seroprevalence of *L. intracellularis* antibody among swine herds in both China and other countries have demonstrated a higher seropositivity in old pigs than in young pigs (16, 24). These findings suggest that old pigs might be at higher risk compared to young pigs.

However, this study also has several limitations. The strict biosecurity actions implemented in pig farms for the purpose of African Swine Fever control increases the difficulty of including more farms in more regions for investigation. Therefore, the survey conducted in this study is a snapshot survey and the data given by this study may not reflect the overall prevalence of *L. intracellularis* on pig farms across China. In addition, a combining use of PCR tests and serological investigation may help to make more solid conclusions. However, collecting blood samples is also not easy at this period. Despite of these limitations, this study still provides insights on the prevalence of *L. intracellularis* on Chinese pig farms, and in the next we intend to conduct a more comprehensive investigation on more farms located on more geographical regions.

As of October 7th, 2022, there are only eight assembled genome sequences of *L. intracellularis* in NCBI Genome database (3), and none of them is from China. The lack of such information may limit the understanding of genomic characteristics of *L. intracellularis* prevalent in pigs in China. Among the eight assembled genome sequences publicly available, two genome sequences were complete [isolate N343 from a sow (25) and isolate PHE/MN1-00 from a pig; both of them were isolated in Minnesota, United States (US)]. In this study, we generated the first full genome sequence of *L. intracellularis* from China. Interestingly, the overall genomic characteristics of the Chinese isolate, including the numbers of plasmids, the sizes of the chromosome and the plasmids, the average GC content, and the numbers of predicted genes, were similar to those of the two US isolates. In addition, the genome sequence of the Chinese isolate was highly identical to those of the two US isolates. These findings suggest that *L. intracellularis* prevalent in China may genetically be related to those prevalent in the US. A possible reason to explain this result might be the frequent import of breeding pigs of China from the United States and Europe, and the pig genetics, bacterial populations and other pig husbandry features of China may therefore largely the same as elsewhere in the global pig industry (26). Indeed, a recent study has demonstrated that *L. intracellularis* isolates associated with disease of pigs in different geographical regions in the world belong to a genetically monomorphic clonal lineage (27).

## Conclusion

In summary, we developed a TaqMan qPCR for the detection of *L. intracellularis* by targeting the bacterial 16S rDNA gene. Using the TaqMan qPCR developed herein, we investigated the prevalence of *L. intracellularis* in pigs belonging to different ages on 47 farms in China. The investigation results indicated a high prevalence rate of *L. intracellularis* on Chinese pig farms. We also generated the first complete genome sequence of *L. intracellularis* from China, and sequence comparisons showed that the genome sequence of the Chinese isolate was highly homologous to those of the field isolates from the United States.

## Methods

### Primer design, standard plasmid construction, and TaqMan qPCR development

To develop a TaqMan qPCR for the detection of *L. intracellularis*, we designed a pair of primers (LI-F: 5′-CGGGATCCCCGATCTAAGAGGATAATC-3′ [*Bam*HI]; LI-R: 5′-CGGAATTCGATCCAAAGACCTTCATC-3′ [*Eco*RI]) based on the bacterial 16S rRNA gene (GenBank accession no. U30147). Genomic DNA of *L. intracellularis* was extracted from a PPE commercial live vaccine (Enterisol^®^Ileitis; Boehringer Ingelheim, St. Joseph, Missouri) using a commercial bacterial DNA preparation kit (TIANGEN, Beijing, China). The target sequence (138 bp) of LI-F/LI-R was amplified from the genomic DNA of *L. intracellularis* through PCR performed in a 25-μl reaction volume containing 1-μl of the template DNA, 1.5-μl of the forward/reverse primer (10 pmol/ul), 12.5-μl of 2×Phanta Flash Master Mix (Vazyme, Nanjing, China), and 8.5-μl nucleotide free water. PCR running conditions were 95°C for 5 min, followed by 35 cycles of 95°C for 30s, 57°C for 35 s, and 72°C for 35s, with a final extension at 72°C for 10 min. PCR product with correct size (138 bp) was confirmed by Sanger sequencing (Supplementary Text 1). Afterwards, the PCR product and the pcDNA3.1 vector were double digested by *Bam*HI/*Eco*RI (TAKARA, Tokyo, Japan), and they were ligated using a DNA ligase (TAKARA, Tokyo, Japan). Finally, the standard plasmid pcDNA-LI (6.73 × 10^10^ copies/μl) was generated by transforming the connected product into *E. coli* DH5α (TAKARA, Tokyo, Japan).

To optimize the amplification conditions for qPCR, the reaction was performed in a 25-μl volume, which contains template DNA 1-μl (2.69 × 10^9^ copies/μl), AceQ^®^ Uniwersal U+ Probe Master Mix (Vazyme, Nanjing, China) 12.5-μl, each of the forward and reverse primers (0.1, 0.2, 0.25, 0.5, 0.75, or 1.0 μM), the TaqMan probe (FAM-CACACTGGAACTGGAACACG-TAMRA; 0.05, 0.1, 0.2, 0.25, or 0.5 μM), and nuclease-free water up to 25-μl. PCR assay was performed on a CFX96 Touch Real-Time PCR Detection System (Bio-Rad, Hercules, CA) with the following conditions: 95°C for 10 min, followed by 40 cycles of 95°C for 15 s, annealing at different temperatures 60°C for 60 s. Fluorescence was recorded at 50°C for 2 min.

To construct the standard curve, a series of 10-fold dilutions of pcDNA-LI (6.73 × 10^9^-6.73 × 10^5^ copies/μl) were used as the template to perform the qPCR assay, which was performed in a 25-μl volume containing 2-μl of the template DNA, AceQ^®^ Uniwersal U^+^ Probe Master Mix (Vazyme, Nanjing, China) 12.5-μl, each of the forward and reverse primers 0.5 μM (final concentration), the TaqMan probe 0.25 μM (final concentration), and nuclease-free water up to 25-μl. PCR was performed on an CFX96 Touch Real-Time PCR Detection System (Bio-Rad, Hercules, CA) with the following conditions: 95°C for 10 min, followed by 40 cycles of 95°C for 15 s, annealing at different temperatures 60°C for 60 s. Fluorescence was recorded at 50°C for 2 min. Standard curves were generated based on the cycle threshold (Ct) values and the copy numbers (lg values) of the template DNA. Coefficients of determination (*R*^2^) were calculated using GraphPad Prism v. 8.0.1 (https://www.graphpad.com/scientific-software/prism/). The experiment was also performed three times independently to verify the repeatability. The detection limit was calculated following the formula *Ct* = −3.547×lg DNA copies + 40.520 (*R*^2^ = 0.9999).

To evaluate the sensitivity, a series of 10-fold dilutions of pcDNA-LI (6.73 × 10^5^-6.73 × 10 copies/μl) were used as the template to perform the qPCR assay. To evaluate the specificity, genomic DNAs of *Escherichia coli* (EC), *Salmonella* (Salm), *Glaesserella parasuis* (GPS), *Brachyspira hyodysenteriae* (Bh), *Streptococcus suis* (Ss), Pseudorabies virus (PRV), Japanese encephalitis virus (JEV), as well as DNAs extracted from intestinal tissues of healthy pigs were used as the templates to perform the qPCR assays. Genomic DNA of *L. intracellularis* and nuclease-free water were used as positive and negative controls, respectively. The efficacy of the qPCR detecting *L. intracellularis* was also compared with that of a qPCR published previously (17).

### Investigation of clinical samples

To investigate the prevalence of *L. intracellularis* in pigs in China, we randomly collected 891 fecal samples from apparently healthy pigs at different ages in 47 farms [seven sow farms (size ranged from 500 sows per farm to 5,000 sows per farm) and 38 fattening farms (size ranged from 600 pigs per farm to 5,000 pigs per farm); Supplementary Table 1] in five provinces located in south China (Guangxi, Guangdong), northeast China (Liaoning), central China (Hubei), and east China (Shanghai). The DNA of *L. intracellularis* in these samples was investigated using the TaqMan qPCR developed in this study. In addition, a total of 705 diarrheal or bloody feces from symptomatic pigs delivered from 125 Chinese farms (Supplementary Table 2) were also investigated. Total DNA was extracted from the samples using a DNA/RNA Extraction Kit (Vazyme, China) and was used as the template DNA for the PCR, which was performed in a 25-μl volume containing 2-μl of the template DNA, AceQ^®^ Uniwersal U^+^ Probe Master Mix (Vazyme, China) 12.5-μl, each of the forward and reverse primers 0.5 μM (final concentration), the TaqMan probe 0.25 μM (final concentration), and nuclease-free water up to 25-μl. PCR was performed on an CFX96 Touch Real-Time PCR Detection System (Bio-Rad, Hercules, CA) with the following conditions: 95°C for 10 min, followed by 40 cycles of 95°C for 15 s, annealing at different temperatures 60°C for 60 s. Fluorescence was recorded at 50°C for 2 min.

### Next-genetration sequencing and bioinformatical analysis

To generate the complete genome sequence of *L. intracellularis*, the ileal sample collected from a pig at 120 days of age died from *L. intracellularis* infection on a farm in Guangxi Province in China was selected for DNA extraction using a DNA/RNA Extraction Kit (Vazyme, China). After quantification using the TBS-380 fluorometer (Turner BioSystems Inc., Sunnyvale, CA), DNA with high quality (OD260/280 = 1.8–2.0, >6 ug) was used for sequencing library preparation. The complete genome sequence of *L. intracellularis* PPE-GX01-2022-Li was sequenced using a combination of PacBio RS and Illumina sequencing platforms. For Illumina sequencing, DNA libraries constructed using an Illumina TruSeq™ Nano DNA Sample Prep Kit following the manufacturer's recommendations were sequenced on an Illumina NovaSeq 6000 platform. We also generated 20 kb insert whole genome shotgun libraries which were sequenced on a Pacific Biosciences RS instrument using standard methods. Briefly, an aliquot of 8 μg DNA was spun in a Covaris g-TUBE (Covaris, MA) at 6,000 rpm for 60 s using an Eppendorf 5424 centrifuge (Eppendorf, NY). DNA fragments were then purified, end-repaired and ligated with SMRTbell sequencing adapters following manufacturer's recommendations (Pacific Biosciences, CA). Resulting sequencing libraries were purified three times using 0.45 × volumes of Agencourt AMPure XPbeads (Beckman Coulter Genomics, MA) following the manufacture's recommendations.

After sequencing, a total of 58,848.9 Mb raw data was generated by the Illumina strategy. The raw paired end reads were trimmed and quality controlled by Trimmomatic (http://www.usadellab.org/cms/uploads/supplementary/Trimmomatic) (28). Following this step, a total of 53,967.8 Mb clean data (Q30: 96.72%) was obtained, and this data was used to evaluate the complexity of the genome and correct the PacBio long reads. PacBio sequencing yielded 17,450,119,530 bp reads (*N*_50_, 8,267 bp; *N*_90_, 6,864 bp) for the samples. Next, the sequence data was assembled using ABySS 2.0 (http://www.bcgsc.ca/platform/bioinfo/software/abyss) (29) with multiple-Kmer parameters, and the PacBio corrected long reads were assembled using Canu (https://github.com/marbl/canu) (30). Subsequently, GapCloser (https://sourceforge.net/projects/soapdenovo2/files/GapCloser/) (31) was applied to fill up the remaining local inner gaps and correct the single base polymorphism for the final assembly results. The complete circle of the genome was drawn with Circos v0.64 (http://circos.ca/) (32).

The complete genome sequence of PPE-GX01-2022 was annotated using RAST Server (33). Gene functions were also annotated against the COG database (http://www.ncbi.nlm.nih.gov/COG) (34) by blastp module. Antimicrobial resistance genes (ARGs) and virulence factor genes (VFGs) were determined by searching the genome sequence against the CARD database (https://card.mcmaster.ca/) (35) and VFDB database (http://www.mgc.ac.cn/VFs/) (36), respectively. Prophages and CRISPR elements were determined by PHASTER (37) and CRISPRfinder (38), respectively. Average nucleotide identities (ANI) between two complete genome sequences of PPE-GX01-2022 and the US isolates N343 (GenBank accession no. NC_020127) and PHE/MN1-00 (GenBank accession no. NC_008011) were calculated using the ANI calculator (http://enve-omics.ce.gatech.edu/ani/) (39). Sequence comparisons were also performed and visualized using EasyFig 2.2.5 (40). Orthologous genes shared by the genomes of PPE-GX01-2022, N343 and PHE/MN1-00, as well as isolate-specific genes possessed by the three isolates were determined using OrthoMCL v.2.0.3 (41) with default parameters. Phylogenetic analysis was performed as previously described (27). To generate a maximum likelihood (ML) tree, the whole genome sequences of *Escherichia coli* strain E2865 (GenBank accession no. AP018808), *Salmonella enterica* strain FDAARGOS_878 (GenBank accession no. CP065719), *L. intracellularis* isolates Fu/JPN (GenBank accession no. QNHO01000000), Ni/JPN (GenBank accession no. QNHN01000000), Ib2/JPN (GenBank accession no. QNHM01000000), CBNU010 (GenBank accession no. JAIPUV010000000), LR189 (GenBank accession no. PRDD01000000), E40504 (GenBank accession no. MTPJ01000000), N343, PHE/MN1-00, and PPE-GX01-2022 were aligned using the MAFFT software (version 7.037) (42) with default parameters. Afterwards, the fasta file obtained from the alignment was input for constructing the ML tree using RAxML (version 8) (43) with the GTR/GAMMA model.

### Statistical analysis

Statistical analysis was performed using “Chi-square test” in the STADA software. *P* < 0.05 was set as result significant.

## Data availability statement

The datasets presented in this study can be found in online repositories. The names of the repository/repositories and accession number(s) can be found at: https://www.ncbi.nlm.nih.gov/genbank/, CP107054; https://www.ncbi.nlm.nih.gov/genbank/, CP107055; https://www.ncbi.nlm.nih.gov/genbank/, CP107056; https://www.ncbi.nlm.nih.gov/genbank/, CP107057.

## Ethics statement

The animal study was approved by Institutional Ethics Committees (IECs) of Huazhong Agricultural University (approval number: HZAUSW-2022-0022). The study was conducted in accordance with the local legislation and institutional requirements.

## Author contributions

LW: Data curation, Funding acquisition, Investigation, Methodology, Writing – original draft. WW: Investigation, Methodology, Writing – review & editing. LZ: Methodology, Writing – review & editing. ZZ: Methodology, Writing – review & editing. XY: Methodology, Software, Writing – review & editing. JF: Methodology, Writing – review & editing. HoC: Methodology, Writing – review & editing. WS: Methodology, Writing – review & editing. XH: Methodology, Writing – review & editing. LH: Methodology, Writing – review & editing. PQ: Supervision, Writing – review & editing. HuC: Supervision, Writing – review & editing. ZP: Funding acquisition, Project administration, Supervision, Writing – original draft, Writing – review & editing. BW: Conceptualization, Funding acquisition, Project administration, Supervision, Writing – review & editing.

## Supplementary material

Supplementary Figure 1 Evaluation of the specificity of the qPCR method developed in this study by generating amplification curves of the method on detecting different pathogens.

Supplementary Table 1 Numbers of fecal samples collected from healthy pigs at different ages in 47 Chinese farms.

Supplementary Table 2 Numbers of fecal samples from diarrheal pigs at different ages in 125 Chinese farms.

Supplementary Table 3 Antimicrobial resistance genes possessed by *L. intracellularis* PPE-GX01-2022.

Supplementary Table 4 Virulence factor genes possessed by *L. intracellularis* PPE-GX01-2022.

Supplementary Table 5 Strain-specific genes possessed by *L. intracellularis* PPE-GX01-2022, PHE/MN1-00, or N343.

Supplementary Text 1 Nucleotide sequence amplified from the genomic DNA of *L. intracellularis* with using the primers designed in this study.
