# Supplementary figures and images for: Fecal PCR survey and genome analysis of Lawsonia intracellularis in China
Source: Front Vet Sci. 2024 Feb 7;11:1324768. doi: 10.3389/fvets.2024.1324768 (PMC10879436; doi:10.3389/fvets.2024.1324768)

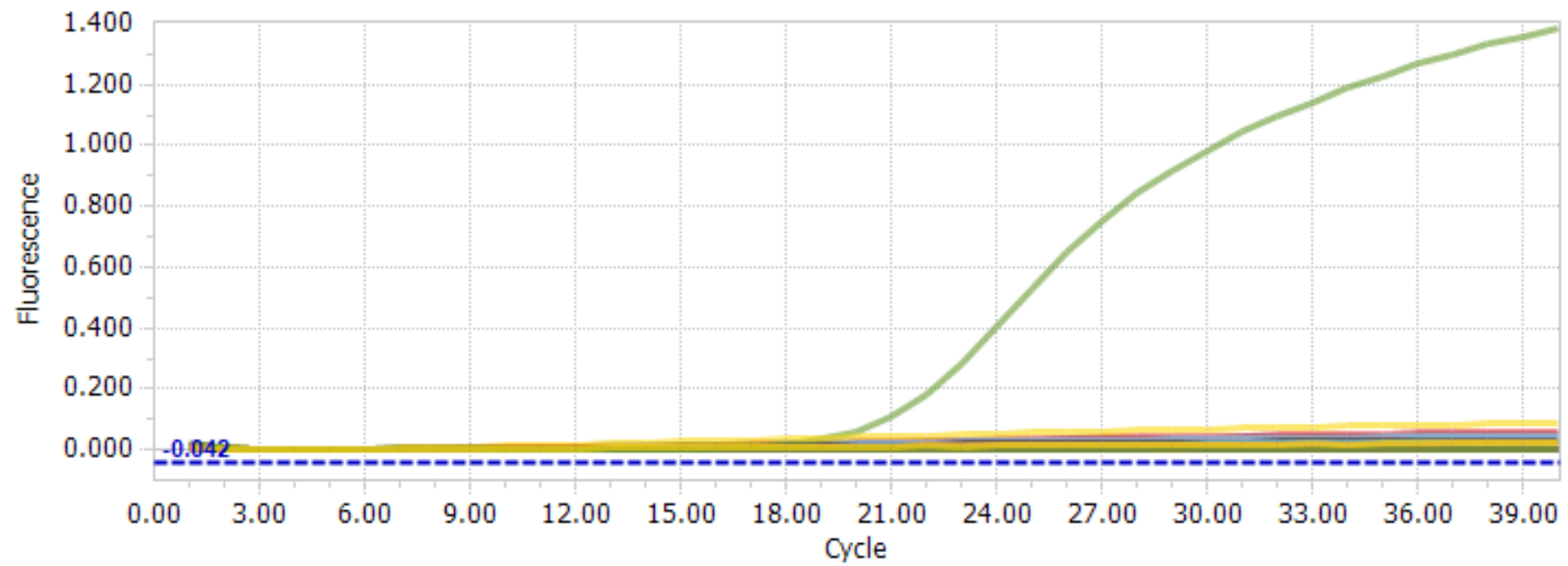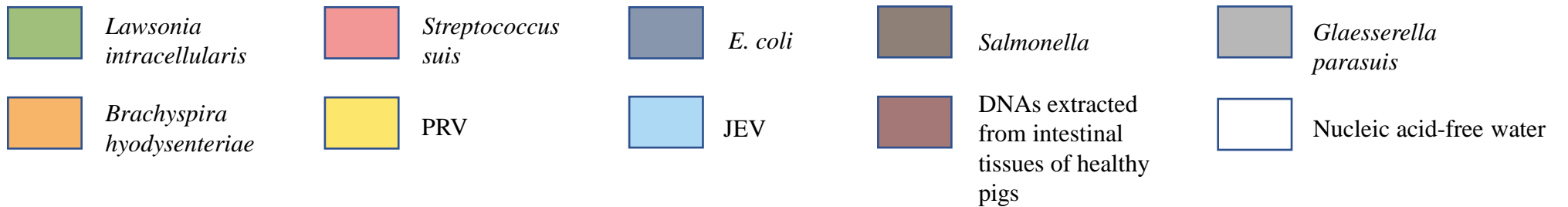

Supplement: Supplementary Figure 1 — Evaluation of the specificity of the qPCR method developed in this study by generating amplification curves of the method on detecting different pathogens. [file Data_Sheet_1.ZIP › Supplementary materials/Fig. S1.pdf]
